# miR-542-3p attenuates corticosterone-induced hippocampal neuronal damage in depressive mice by modulating PTEN/AKT/GSK3β/β-catenin pathway

**DOI:** 10.17305/bb.2025.11523

**Published:** 2025-02-24

**Authors:** Ningbo Yang, Jie Li, Hongxia Hu, Xujiang Wang

**Affiliations:** 1 Department of Psychiatry, First Affiliated Hospital, College of Clinical Medicine of Henan University of Science and Technology, Jianxi, China; 2 Department of Emergency Medicine, The First Affiliated Hospital of Henan University of Science and Technology, Luolong, China; 3 Department of Clinical Laboratory Medicine, The First Affiliated Hospital of Henan University of Science and Technology, Jianxi, China; 4 Centre for Psychological Heath Education of Henan University of Science and Technology, Luolong, China

**Keywords:** miR-542-3p, phosphatase and tensin homolog, PTEN, AKT/GSK3β/β-catenin pathway, hippocampal neuronal damage

## Abstract

Depression is a common psychological disease, and nerve injury is the key link of depression. The molecular mechanism involved in this link needs to be explored. miR-542-3p can reduce the degree of hippocampal neuronal damage in rats, but its mechanism in the neural damage of depression is still unclear. HT-22 cell injury was induced by corticosterone (CORT). After overexpression or knockdown of miR-542-3p, CORT-induced HT-22 cell injury was tested by cell counting kit-8 (CCK-8) assay, lactate dehydrogenase (LDH) assay and flow cytometry. Inflammatory and oxidative stress indicator levels were analyzed by kit and flow cytometry. The target genes of miR-542-3p were obtained by database analysis, and the targeting relationship between miR-542-3p and phosphatase and tensin homolog (PTEN) was explored based on dual luciferase assay. After PTEN overexpression or application of AKT pathway agonist MK-2206, the degree of cell damage, inflammation, and oxidative stress were detected again. CORT was used to induce depression in mice. Pathological changes of brain tissue structure and neuronal survival were observed by pathological staining. The miR-542-3p, PTEN, and AKT/GSK3β/β-catenin pathway protein levels *in vivo* and *in vitro* were detected by qRT-PCR and Western blot. Overexpression/knockdown of miR-542-3p alleviated/aggravated CORT-induced cell injury, inflammation, and oxidation levels in HT-22 cells (*P* < 0.05). Meanwhile, overexpressed miR-542-3p can reduce neurological damage of mice. miR-542-3p can target PTEN, and it can trigger the AKT/GSK3β/β-catenin pathway by targeting PTEN expression to reduce CORT-induced nerve injury (*P* < 0.05). miR-542-3p can reduce CORT-induced hippocampal neuronal damage by targeting PTEN and activating the AKT/GSK3β/β-catenin pathway.

## Introduction

Depression is a common mental illness characterized by anxiety, fatigue, slowed thinking, cognitive decline, and an increased risk of self-harm or even suicide. It not only affects a patient’s well-being but also has a negative impact on their family life and overall safety [1, 2]. Depression can arise from a combination of physiological, psychological, and environmental factors. While commonly used antidepressant medications can be effective, they also come with challenges, such as drug resistance and various adverse side effects [3].

Beyond its psychological and emotional toll, depression also causes significant damage to the brain, affecting both its structure and function. Studies suggest that depression and nerve injury are mutually causal—depression is linked to neurological dysfunction, with neuronal damage playing a key role. Imaging studies have shown that individuals with depression exhibit decreased hippocampal density, reduced dendritic branching, and neuronal necrosis [4]. Similarly, animal models of depression also show hippocampal neuron damage [5]. Therefore, targeting neuronal damage and investigating its underlying mechanisms could provide valuable insights into potential treatments for depression.

MicroRNA (miRNA) is a small, non-coding RNA that has become a key focus in nerve cell research [6]. miR-542-3p is normally expressed in brain tissue, but its levels are reduced in human glioma cells, where it plays a role in inhibiting cell migration and invasion [7]. Research has shown that miR-542-3p is downregulated in the hippocampal neurons of epileptic rats, as confirmed through both experimental and clinical studies. Notably, increased expression of miR-542-3p can not only reduce hippocampal neuron damage in epileptic rats but also lower the frequency of epileptic seizures and decrease apoptosis [8]. Additionally, upregulating miR-542-3p inhibits NLRP3-mediated inflammatory activation and reduces neuronal pyroptosis [9]. Given these findings, we propose that miR-542-3p has neuroprotective effects and may also help prevent hippocampal neuron damage in depression. This potential role offers valuable insights into the molecular mechanisms underlying depression and provides a new direction for its treatment.

Phosphatase and tensin homolog (PTEN) is a lipid and protein phosphatase that regulates cell growth and survival. It primarily acts as a tumor suppressor, and its proper localization is crucial for its function [10]. Nuclear localization of PTEN plays a key role in cell growth regulation and can promote cell survival [11]. While PTEN is strongly associated with cancer syndromes, it also plays an essential role in neurodevelopment. Mutations in the PTEN gene can lead to focal abnormalities in white matter, which may contribute to anxiety disorders and developmental delays [12]. PTEN is also implicated in the development of autism and other neurodevelopmental disorders. It can exacerbate oxidative damage and promote hippocampal cell apoptosis [13], while neuronal dysplasia has been linked to depression [14, 15]. Based on this, it can be speculated that PTEN may help alleviate depression by mitigating neuronal damage.

PTEN can inhibit the PI3K/AKT pathway, influencing the signaling cascade’s progression [16]. AKT, a serine/threonine kinase, plays a key role in cell survival, differentiation, and movement. Its phosphorylation activates GSK3β, which facilitates the proper assembly of microtubules in neurites. This process, however, can lead to excessive branching, ultimately disrupting synaptic connections in adult-born granule neurons [17]. β-catenin, the primary substrate of GSK3β, has recently been identified as a crucial factor in behavioral recovery [18]. Studies show that selectively knocking out β-catenin in the brain promotes depression-like behavior under chronic stress conditions [19]. Conversely, antidepressants can upregulate β-catenin in the hippocampus of depressed rats, promoting neurogenesis [20]. By inhibiting GSK3β activity, β-catenin stabilization is enhanced, thereby supporting neurogenesis and contributing to antidepressant effects [21]. In summary, we propose that PTEN may alleviate neurological damage associated with depression through the AKT/GSK3β/β-catenin pathway.

It remains unclear whether miR-542-3p can target PTEN and mediate this signaling pathway to regulate nerve injury in depression, making it a topic worth exploring. Based on this, we hypothesize that miR-542-3p and PTEN are involved in the pathogenesis of corticosterone (CORT)-induced neuronal damage in depressed mice. To investigate this, we use CORT-induced hippocampal neuronal damage models to examine changes in miR-542-3p expression, analyze its target genes and regulatory pathways, and assess the impact of these changes on molecular expression levels and neuronal damage. This study aims to provide new insights for identifying molecular targets in the clinical treatment of depression.

## Materials and methods

### Cell culture and processing

Mouse hippocampal neuron cell line HT-22 was obtained from the Cell Bank of the Chinese Academy of Sciences (Shanghai, China) and cultured in DMEM medium supplemented with 10% fetal bovine serum (C0235, Beyotime, Shanghai, China) at 37 ^∘^C in a 5% CO_2_ atmosphere.

PTEN overexpression (PTEN) and its negative control (vector), miR-542-3p overexpression (mimics) and its negative control (mimics NC), as well as miR-542-3p knockdown (inhibitor) and its negative control (inhibitor NC), were purchased from RiboBio (Guangzhou, China). The cells were then transfected using Lipofectamine 3000 (L3000001, Invitrogen, Austin, TX, USA), with all transfections performed for 48 h.

HT-22 cells were divided into five groups: Control, Model, miR-542-3p overexpression/knockdown, miR-542-3p overexpression + PTEN overexpression (mimics + PTEN), and miR-542-3p overexpression + AKT inhibitor MK-2206 (mimics + MK-2206). The Control and Model groups were cultured in DMEM for 24 h, while the other groups were transfected with miR-542-3p and PTEN. Additionally, the mimics + MK-2206 group was treated with 1 µM MK-2206. Except for the Control group, all groups were exposed to 200 µM CORT for 24 h to induce injury.

### The effect of CORT on the viability of HT-22 cells

HT-22 cells in the logarithmic growth phase were seeded into 96-well plates at 100 µL per well and incubated for 24 h. The cells were then divided into a control group and an experimental group. The control group was cultured in complete medium, while the experimental group was treated with CORT at concentrations of 50, 100, 200, 250, and 500 µM. After 24 h of incubation, 10 µL of cell counting kit-8 (CCK-8) working solution (CA1210, Solarbio) was added to each well and gently mixed. The plates were then incubated for an additional 2 h in a cell incubator. Absorbance was measured at 450 nm using a microplate reader to determine OD values, assess cell viability, and identify the optimal CORT concentration.

### qRT-PCR assay

HT-22 cells were treated with 50, 100, 200, 250, and 500 µM CORT for 24 h. Total RNA was extracted using the TransZol Up reagent (ET111-01-V2, TRANS, Beijing, China), while miRNA was isolated using the EasyPure miRNA Kit (ER601-01-V2, TRANS). RNA concentration and quality were assessed using a nucleic acid analyzer (Q5000, Quawell, Beijing, China), with OD260/280 nm ratios ranging from 1.8 to 2.0. For cDNA synthesis, AMV reverse transcriptase (2621, TAKARA, Tokyo, Japan) was used for reverse transcription. PCR amplification was performed using TB Green FAST qPCR (CN830S, TAKARA). Additionally, TransScript Green miRNA Two-Step qRT-PCR SuperMix (AQ202-01, TRANS) was used to synthesize first-strand cDNA from miRNA, followed by PCR amplification. Dual-distilled water served as a template-free negative control (NC) to monitor potential contamination. The cycle threshold (Ct) value, representing the number of cycles required for the fluorescence signal to reach a specific threshold, was used for data analysis. The relative expression levels of mRNA and miRNA were calculated using the 2^−ΔΔCt^ method, with *U6* and *GAPDH* serving as internal controls.

The primer sequences: *miR-542-3p*: F: 5′-GCGCGATATCGCGACGAGCGACC-3′; R: 5′-TTAAGCGAGCTATCGCGCGCGAGCG-3′; *PTEN*: F: 5′-TGAGTTCCCTCAGCCATTGCCT-3′; R: 5′-GAGGTTTCCTCTGGTCCTGGTA-3′; *U6*: F: 5′-GTTCAGGAAGAGTGACACCA-3′; R: 5′-TTCTCCGCATCTCCATTCTC-3; *GAPDH*: F: 5′-AATGGATTTGGACGCATTGGT-3′; R: 5′-TTTGCACTGGTACGTGTTGAT-3′. The above primers were provided and purified by Sangon Biotech (Shanghai, China).

### Bioinformatics analysis

The potential targets of miR-542-3p were identified using the miRWalk (http://129.206.7.150/), TargetScan (https://www.targetscan.org/vert/_80/), miRDB (https://mirdb.org/), and ENCORI/starBase (https://rnasysu.com/encori/) databases. TargetScan specifically predicted the binding region between miR-542-3p and PTEN.

### Double luciferase assay

The 3′-UTR fragment containing the binding region of miR-542-3p and PTEN was cloned into a plasmid vector for transformation. Plasmids incorporating the miR-542-3p and PTEN binding regions were designed with both wild-type (WT) and mutant (MUT) sequences. Using the Lipofectamine 2000 kit (Invitrogen Inc., Carlsbad, CA, USA), 200 ng of plasmid and 30 nM of miR-542-3p mimics or mimic NC were co-transfected and incubated at 37 ^∘^C for 24 h. Fluorescence intensity was measured using a dual-luciferase detection system (Promega Corporation, Madison, WI, USA).

### Lactate dehydrogenase (LDH) test

The LDH cytotoxicity assay kit (C0016, Beyotime, Shanghai, China) was used to assess cell damage. HT-22 cell suspensions were seeded into 96-well plates at a density of 1 × 10^4^ cells/well. After 24 h, the model was established, and treatments were administered. The experiment included blank control wells (cell-free culture medium) and untreated control wells to measure the sample’s maximum enzyme activity. One hour before the experiment concluded, 20 µL of LDH reagent was added to the maximum enzyme activity control wells, mixed thoroughly, and incubated in the cell culture chamber until the end of the experiment. After centrifugation, 120 µL of the supernatant was collected and transferred to a new culture plate. The OD value was measured at a wavelength of 490 nm using an automatic microplate reader.

### Detection of apoptosis by flow cytometry

Apoptosis was assessed using the Annexin V-FITC/PI Apoptosis Detection Kit (CA1020, Solarbio). HT-22 cells subjected to different treatments were collected and washed with PBS. Approximately 1 × 10^6^ cells were obtained for each group and resuspended in 500 µL of Binding Buffer. Then, 5 µL of Annexin V-FITC and 10 µL of PI were added, and the mixture was incubated in the dark for 15 min. The samples were then transferred to flow cytometry loading tubes, and apoptosis was analyzed using a BD FACSCalibur™ flow cytometer (BD Biosciences, San Jose, CA, USA) within 1 h. The apoptosis rate was subsequently calculated.

### Determination of antioxidant enzyme levels

HT-22 cells were collected after different treatments and subjected to ultrasonic crushing. The supernatant was then obtained by centrifugation. The levels of antioxidant enzymes were measured using a Superoxide Dismutase (SOD) Assay Kit and a Malondialdehyde (MDA) Assay Kit (A001-3-2, A003-1-2, Jiancheng Bioengineering Institute, Nanjing, China), following the manufacturer’s instructions. The optical density (OD) was measured at 450 nm to determine the enzyme content in each group.

### Reactive oxygen species (ROS) level

The ROS Detection Kit (CA1410, Solarbio) was used to measure ROS levels. HT-22 cells were collected and adjusted to a concentration of 1 × 10^6^/mL. DCFH-DA was added at a 1:1000 ratio to achieve a final concentration of 1 µmol/L. The cells were incubated for 30 min, then stimulated with ROS for another 30 min. ROS levels were subsequently analyzed using flow cytometry.

### Detection of inflammatory factors by ELISA

Interleukin (IL)-6 (SEKM-0007), IL-1β (SEKM-0002), and tumor necrosis factor-α (TNF-α, SEKM-0034) ELISA kits were purchased from Solarbio. After the experiment, HT-22 cells were collected and centrifuged, and the supernatant was then collected. IL-6, IL-1β, and TNF-α levels were measured according to the manufacturer’s instructions.

### Animal grouping and processing

The depression-like behavior model in male mice was induced using CORT [22]. All mice were randomly divided into five equal groups (*n* ═ 8 per group): Control, Model (CORT), miR-542-3p Overexpression Negative Control (agomiR NC), miR-542-3p Overexpression (agomiR-542-3p), and miR-542-3p Overexpression + AKT Inhibitor MK-2206 (agomiR-542-3p + MK-2206). Mice in the control group received daily subcutaneous injections of 0.9% normal saline, while those in the other groups were injected with 20 mg/kg CORT. Additionally, mice in the agomiR NC, agomiR-542-3p, and agomiR-542-3p + MK-2206 groups received 5 nmol of agomiR NC or agomiR-542-3p via lateral ventricle injection once per week. The procedure was as follows: after anesthesia with 2% isoflurane, mice were fixed on a brain stereotaxic instrument, their skin was disinfected, and a 0.5 cm midline incision was made on the head. A microsyringe was used to inject 2 µL of the corresponding solution into the lateral ventricle at a depth of 3 mm, positioned 2.5 mm posterior to the anterior fontanel and 2 mm to the right. After the injection, the needle was held in place for 5 min before being slowly withdrawn. The wound was then sutured and disinfected. Once the mice regained consciousness, they were returned to their cages for continued feeding. In addition, the agomiR-542-3p + MK-2206 group received a daily oral dose of 120 mg/kg MK-2206 (Beyotime, Shanghai, China) for 21 days. Following the completion of behavioral testing, the mice were anesthetized and euthanized by cervical dislocation. Whole-brain tissue was collected, washed with normal saline, and the hippocampus was carefully dissected on ice. The hippocampal tissue was then stored at –80 ^∘^C for further analysis.

### Observation of depressive behavior

The sucrose preference test (SPT), tail suspension test (TST), and forced swimming test (FST) were used to assess changes in depressive behavior in mice. Two evaluators, blinded to the experimental groups, conducted the assessments. The SPT followed the method described by Gu [23], measuring sucrose solution and pure water consumption after 12 h. The TST and FST were performed according to the method of Sun [24], recording the total time the mice spent in suspension and in water.

### HE staining

The mouse brain tissue was fixed with 4% paraformaldehyde, embedded in paraffin, and sectioned into 4 µm thick slices. These sections were stained with hematoxylin (C0107, Beyotime) for 15 min, differentiated in 1% acidic alcohol (containing 70% hydrochloric acid) for 30 s, rinsed with running water, and then stained with 0.5% eosin (G1100, Solarbio) for 3 min. The slices were then dehydrated through an alcohol gradient, cleared with xylene, and sealed with neutral gum (G8590, Solarbio). Finally, the hippocampal CA1 region was examined under a microscope.

### Nissl staining

Nissl bodies were stained using the Nissl staining kit (G1434, Solarbio). Brain tissue sections were incubated with methylene blue for 10 min, followed by differentiation in the designated medium for 1 min. The sections were then treated with ammonium molybdate solution for 3 min and mounted using neutral gum (G8590, Solarbio). The number and morphological changes of Nissl bodies in the hippocampal CA1 region were observed under a microscope.

### TUNEL/NeuN immunofluorescence

Brain tissue sections were dewaxed with xylene, rehydrated, and subjected to antigen retrieval. The sections were then blocked with a mixed solution of 5% BSA and 0.5% Triton X-100 for 2 h. Rabbit anti-NeuN (ab177487, 1:1000, Abcam) was added and incubated overnight at 4 ^∘^C, followed by a 2-h incubation with goat anti-rat IgG (GB21302, 1:500, Servicebio, Wuhan, China). Next, a 20 µg/mL proteinase K solution (without DNase) was applied for 30 min. Apoptotic cells were labeled with 50 µL of TUNEL detection solution (C1091, Beyotime) for 60 min in the dark, followed by DAPI staining for 5 min. Finally, the sections were sealed with an anti-fluorescence quenching solution, and the hippocampal CA1 area was observed and imaged using a fluorescence microscope (Olympus VS200, Shinjuku, Tokyo).

### Western blot

After various treatments, HT22 cells and mouse hippocampus tissue were collected, and the supernatant was fully lysed. The total protein concentration was measured using a BCA quantification kit (PC0020, Solarbio). All samples were separated by electrophoresis, and the proteins were then transferred onto PVDF membranes (YA1700, Solarbio).

The membranes were blocked with 5% skimmed milk powder (LP0033B, Solarbio) for 2 h and then incubated overnight at 4 ^∘^C with the following primary antibodies: PTEN (ab267787, 1:1000, Abcam), APBB2 (ab137888, 1:1000), AKT (ab8805, 1:1000), GSK3β (ab93926, 1:1000), β-catenin (ab32572, 1:5000), PSD95 (ab238135, 1:2000), SYP (ab32127, 1:20000), p-GSK3β Ser9 (ab75814, 1:1000), Bax (ab32503, 1:2000), p-AKT Thr308 (ab38449, 1:1000), Bcl-2 (ab182858, 1:2000), and GAPDH (TA-08, 1:1000, ZSGB-BIO, Beijing, China). After incubation, the membranes were washed with TBST buffer (T1082, Solarbio) and incubated with a secondary antibody (1:20,000) for 1 h. Following five washes with TBST buffer, ECL reagent (PE0010, Solarbio) was applied for 2–3 min. Protein signals were then detected using an automatic chemiluminescence imaging system.

### Ethical statement

Forty male C57/BL6 mice (SPF grade), aged six weeks and weighing (20 ± 2) g, were obtained from Spyford Biotechnology Co., Ltd. (Beijing, China). The mice were housed under standard conditions (temperature: 20 to –24 ^∘^C, relative humidity: 50%–70%, light/dark cycle: 12h/12h). This study was approved by the Animal Ethics Committee of The First Affiliated Hospital of Henan University of Science and Technology (Approval No. HK-20230417).

### Statistical analysis

Three independent biological replicates were used. The Shapiro–Wilk test was conducted to assess the normality of the measurement data. All measurement data followed a normal distribution and were expressed as mean ± standard deviation. Statistical analysis and image generation were performed using GraphPad 9.0. One-way ANOVA was used to compare multiple groups. For pairwise comparisons, the least significant difference (LSD) test was applied when variances were homogeneous, while the Games–Howell test was used when variances were heterogeneous. Non-normally distributed data were analyzed using a non-parametric test. A *P* value of < 0.05 was considered statistically significant. When significant differences were found, the Tukey method was used for post hoc analysis.

## Results

### miR-542-3p attenuates CORT-induced HT-22 cell injury in mouse hippocampal neurons

**Figure 1.**
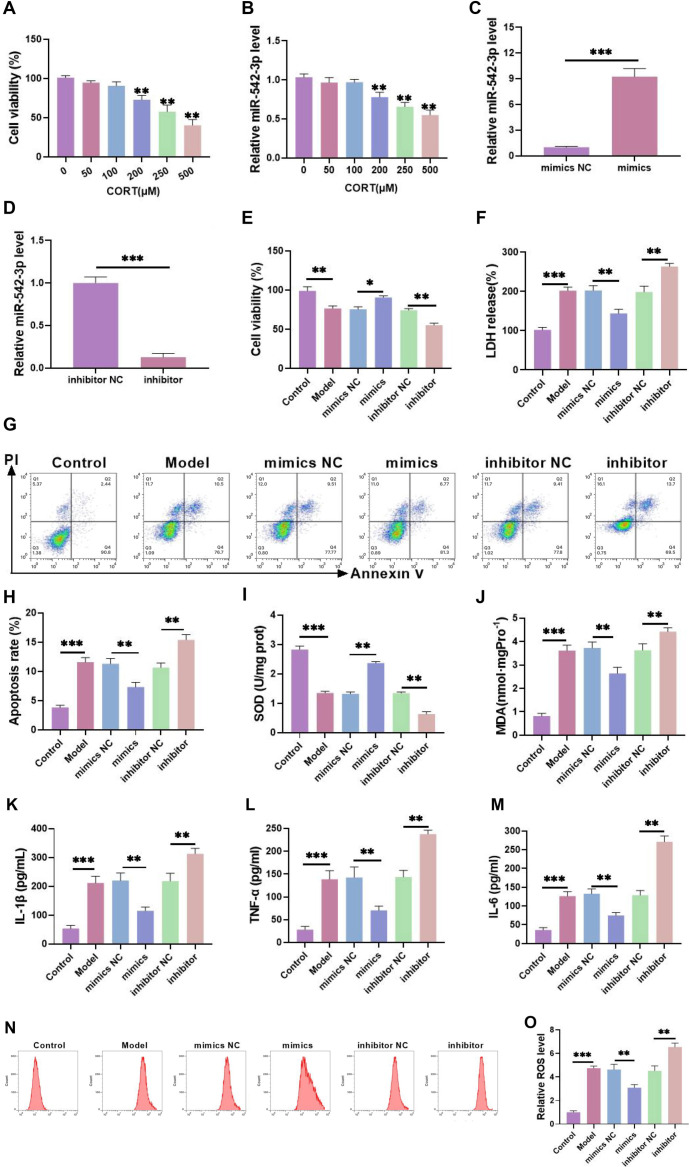
**miR-542-3p attenuates CORT-induced HT-22 cell injury in mouse hippocampal neurons.** (A) The cell survival rate was tested by CCK-8 after various levels of CORT treatment for 24 h. It was found that the cell survival rate began to decrease significantly after 200 µM CORT treatment; (B) *miR-542-3p* level was tested by qRT-PCR after various levels of CORT treatment for 24 h. The level of *miR-542-3p* began to decrease obviously after 200 µM CORT treatment; (C and D) The overexpression/knockdown efficiency of *miR-542-3p* was tested by qRT-PCR, and it was effectively overexpressed/knocked down; (E) The proliferation of HT-22 cells was tested by CCK-8 under various treatment conditions. It was found that the cell viability was markedly decreased after CORT intervention, and it was significantly increased/decreased after miR-542-3p overexpression/knockdown; (F) The damage degree of HT-22 cells treated with various treatments was detected. It was found that the release of LDH was markedly raised after CORT treatment, and it was notably declined/increased after overexpression/knockdown of miR-542-3p; (G and H) The apoptosis rate was notably elevated after CORT intervention, and it was obviously declined/increased after miR-542-3p overexpression/knockdown; (I) The content of SOD was detected by ELISA. The content of SOD was markedly reduced after CORT intervention, and the it was markedly increased/reduced after miR-542-3p overexpression/knockdown; (J) The content of MDA was detected by ELISA. MDA content was evidently enhanced after CORT treatment, and it was evidently decreased/enhanced after miR-542-3p overexpression/knockdown; (K–M) The TNF-α, IL-1β, and IL-6 levels were tested by ELISA. They were considerably elevated after CORT treatment, and they were considerably decreased/raised after miR-542-3p overexpression/knockdown; (N and O) The level of ROS was tested by flow cytometry. It was found that ROS level was considerably boosted after CORT treatment, and it was considerably reduced/increased after overexpression/knockdown of miR-542-3p. *n* ═ 3, ^*^P < 0.05, **P < 0.01, ***P < 0.001. PTEN: Phosphatase and tensin homolog; CCK-8: Cell counting kit-8; ELISA: Enzyme-linked immunosorbent assay; SOD: Superoxide dismutase; MDA: Malondialdehyde; ROS: Reactive oxygen species; CORT: Corticosterone; qRT-PCR: Quantitative real-time polymerase chain reaction; NC: Negative control.

Firstly, we used CCK-8 and qRT-PCR assays to assess cell survival and *miR-542-3p* expression levels after 24 h of treatment with various concentrations of CORT. The cell survival rate began to decrease significantly following exposure to 200 µM CORT (Figure 1A), and *miR-542-3p* expression also showed a significant decline (Figure 1B). Based on these results and previous studies, we selected 200 µM CORT for subsequent experiments.

To investigate the role of *miR-542-3p* in CORT-induced injury in HT-22 cells, we overexpressed or knocked down *miR-542-3p* and confirmed transfection efficiency. The *miR-542-3p* expression level was significantly increased in the overexpression group and decreased in the knockdown group (Figure 1C and 1D), confirming successful transfection. Overexpression of miR-542-3p significantly improved cell viability following CORT treatment, while knockdown further exacerbated CORT-induced damage (Figure 1E), suggesting that miR-542-3p plays a protective role against CORT-induced cytotoxicity.

LDH is widely used as a marker of cytotoxicity. CORT treatment significantly increased LDH activity, whereas miR-542-3p overexpression notably reduced LDH levels. Conversely, knockdown of miR-542-3p further increased LDH activity (Figure 1F), indicating that miR-542-3p mitigates CORT-induced cell damage. Next, we assessed apoptosis rates. CORT treatment significantly increased apoptosis, whereas miR-542-3p overexpression reduced apoptosis, and knockdown further elevated apoptotic rates (Figure 1G and 1H). These findings suggest that miR-542-3p regulates CORT-induced neuronal injury by enhancing cell viability and reducing apoptosis. Inflammation and oxidative stress play critical roles in neuronal cell death [25]. SOD and MDA are commonly used markers of oxidative stress. CORT treatment significantly reduced SOD levels and increased MDA levels. Overexpression of miR-542-3p restored SOD levels and decreased MDA content, while knockdown had the opposite effect (Figure 1I and 1J). The levels of TNF-α (Figure 1K), IL-1β (Figure 1L), and IL-6 (Figure 1M), as well as ROS intensity (Figure 1N and 1O), followed the same trend as MDA, further supporting the notion that miR-542-3p inhibits CORT-induced inflammation and oxidative stress in HT-22 cells. These protective effects may be key mechanisms underlying miR-542-3p’s role in neuronal protection.

### miR-542-3p targeted regulation of PTEN

**Figure 2.**
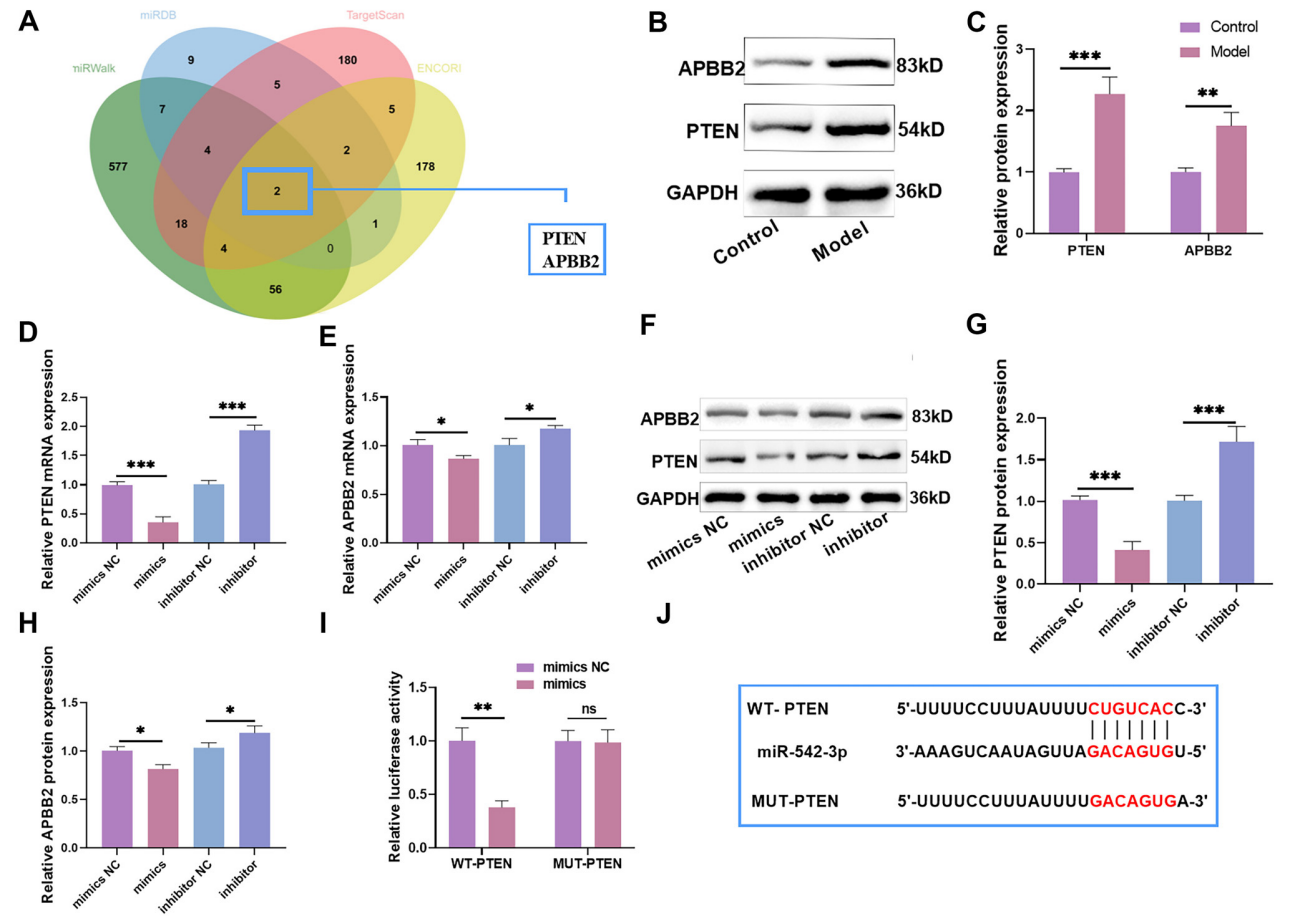
**miR-542-3p targeted regulation of PTEN.** (A) The potential target *PTEN* and *APBB2* of *miR-542-3p* were obtained by the intersection of miRWalk, TargetScan, mirRDB, ENCORI/starBase database; (B and C) Western blot showed that PTEN and APBB2 levels were notably elevated after CORT treatment, but the increase effect of APBB2 was not as good as that of PTEN; (D–H) *PTEN* and *APBB2* levels were notably declined/increased after miR-542-3p overexpression/knockdown, but the expression of *PTEN* was more notable; (I) WT-PTEN and MUT-PTEN luciferase reporter plasmids were co-transfected with mimics NC and mimics into HT-22 cells, respectively. The luciferase activity was tested. Overexpression of miR-542-3p markedly inhibited the luciferase activity of WT-PTEN; (J) The binding sequence of miR-542-3p and PTEN. *n* ═ 3, ^*^P < 0.05, **P < 0.01, ***P < 0.001. PTEN: Phosphatase and tensin homolog; APBB2: Amyloid beta precursor protein binding family B member 2; CORT: Corticosterone; NC: Negative control; WT: Wild-type; MUT: Mutant.

Bioinformatics analysis showed that when the miRWalk targeting score was ≥0.9, the miRDB score was ≥ 90, and the ENCORI/starBase score was ≥1.5, the potential targets of *miR-542-3p* were identified as *PTEN* and *APBB2* (Figure 2A). This suggests that *miR-542-3p* could regulate *PTEN* and *APBB2*. Next, we measured PTEN and APBB2 expression levels and found that both were significantly increased after CORT treatment. However, the upregulation of APBB2 was less pronounced than that of PTEN (Figure 2B and 2C), indicating that PTEN may play a more prominent role in nerve injury. Overexpression or knockdown of miR-542-3p led to a significant decrease or increase in *PTEN* and *APBB2* expression, respectively, with a stronger effect observed for *PTEN* (Figure 2D–2H). Since PTEN exhibited a more robust response and APBB2 was not significantly expressed following CORT treatment, we selected PTEN for further experiments. To confirm whether miR-542-3p directly binds to PTEN, we performed a luciferase reporter assay. Overexpression of miR-542-3p significantly inhibited the luciferase activity of WT-PTEN (Figure 2I), confirming a direct interaction between miR-542-3p and PTEN. The TargetScan database analysis further supported this interaction, showing a context++ score of −0.20, a context++ score percentile of 88, and a weighted context++ score of −0.19. The binding sequence is illustrated in Figure 2J. In summary, miR-542-3p directly targets and downregulates PTEN.

### miR-542-3p inhibits CORT-induced HT-22 injury in mouse hippocampal neurons by inhibiting PTEN

**Figure 3.**
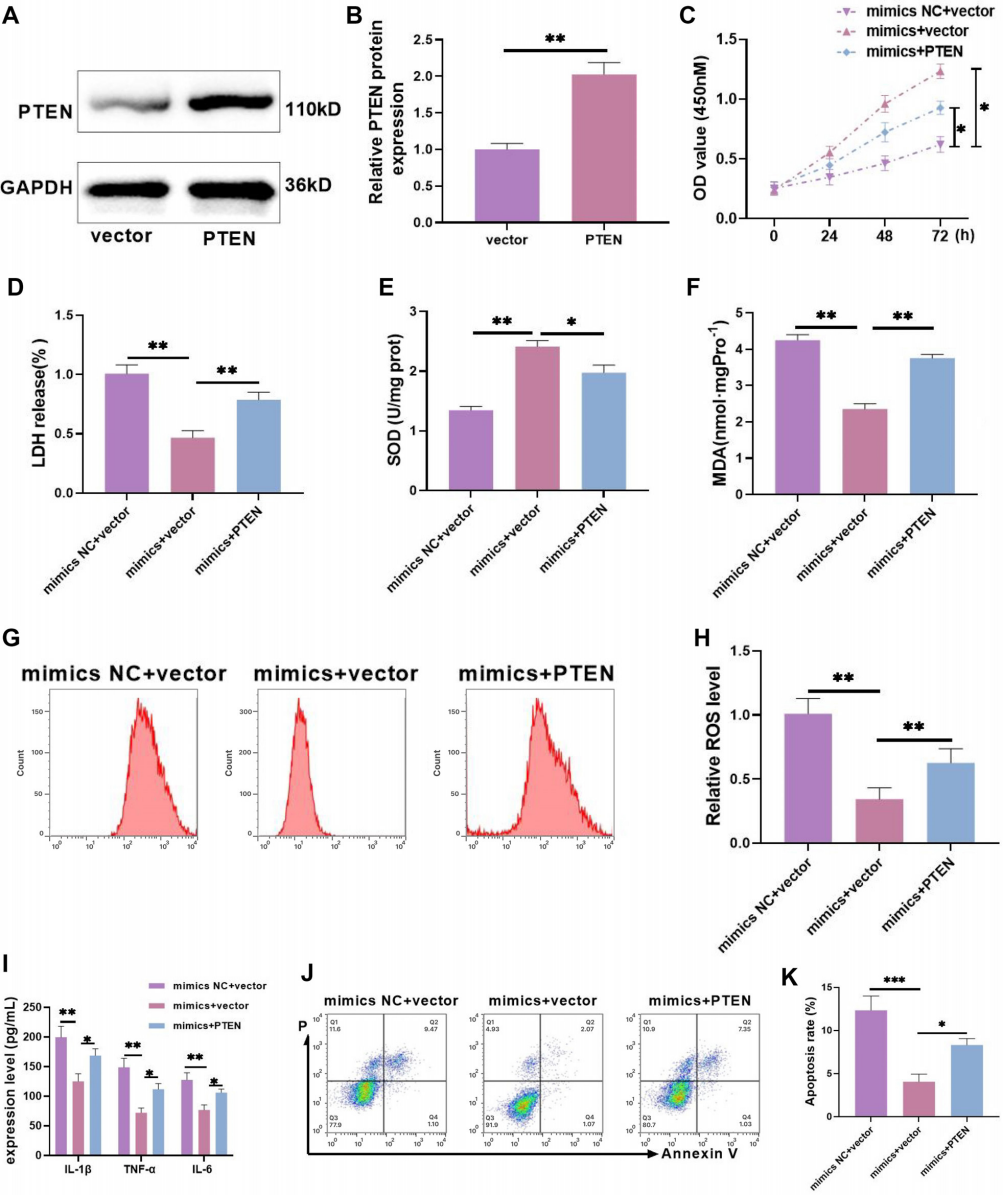
**miR-542-3p inhibits corticosterone-induced HT-22 injury in mouse hippocampal neurons by inhibiting PTEN.** (A and B) The overexpression efficiency of PTEN was tested by Western blot, and PTEN was effectively overexpressed; (C) The proliferation of HT-22 cells under various treatment conditions was tested by CCK-8. Compared with miR-542-3p overexpression alone, the cell viability was notably lower after overexpressed PTEN; (D) LDH was used to test the damage degree in HT-22 cells treated with different treatments. Compared with miR-542-3p overexpression alone, PTEN overexpression notably elevated cell damage degree; (E) The content of SOD was detected by ELISA. Compared with miR-542-3p overexpression alone, it was notably shrunk after PTEN overexpression; (F) The MDA content was detected by ELISA; (G and H) The ROS level was tested by flow cytometry, ROS level was markedly enhanced after PTEN overexpression; (I) The levels of IL-1β, TNF-α, and IL-6 were tested by ELISA, they were significantly increased after PTEN overexpression compared with miR-542-3p overexpression alone; (J and K) The apoptosis was tested by flow cytometry. After PTEN overexpression, the apoptosis rate was markedly higher. *n* ═ 3, ^*^P < 0.05, **P < 0.01. PTEN: Phosphatase and tensin homolog; CCK-8: Cell counting kit-8; ELISA: Enzyme-linked immunosorbent assay; SOD: Superoxide dismutase; MDA: Malondialdehyde; ROS: Reactive oxygen species; IL: Interleukin; TNF-α: Tumor necrosis factor-α; NC: Negative control.

Based on the above results, we further investigated whether miR-542-3p targets PTEN to influence CORT-induced damage in HT-22 cells. First, we verified the efficiency of PTEN overexpression, and Western blot analysis confirmed that PTEN protein was successfully overexpressed (Figure 3A and 3B), indicating that subsequent experiments could proceed. Next, the experiment was divided into three groups: the model group (mimics NC + vector), the miR-542-3p overexpression group (mimics + vector), and the miR-542-3p overexpression + PTEN overexpression group (mimics + PTEN). Consistent with previous findings, miR-542-3p overexpression alleviated inflammatory and oxidative stress-related cell injury and improved cell viability. However, when PTEN was overexpressed, cell viability significantly decreased (Figure 3C), LDH activity markedly increased (Figure 3D), SOD content was significantly reduced (Figure 3E), and MDA levels were notably elevated (Figure 3F). Additionally, ROS intensity (Figure 3G and 3H), as well as TNF-α, IL-1β, and IL-6 levels (Figure 3I) and the apoptosis rate (Figure 3J and 3K), followed the same trend as MDA. These findings indicate that PTEN overexpression significantly counteracted the protective effects of miR-542-3p overexpression against cell injury. Combined with bioinformatics analysis, our results suggest that miR-542-3p targets PTEN to mitigate CORT-induced cell damage, primarily by suppressing oxidative stress and inflammatory responses.

### Overexpression of miR-542-3p negatively regulates PTEN expression to activate AKT/GSK3β/β-catenin

**Figure 4.**
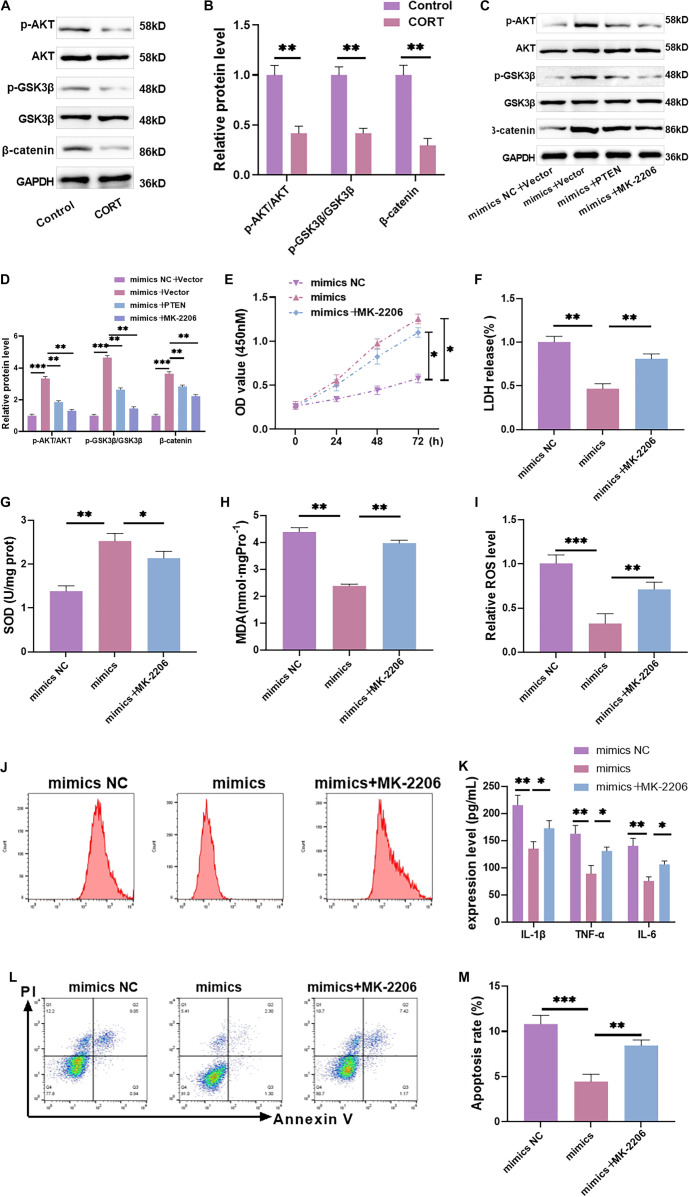
**Overexpression of miR-542-3p negatively regulates PTEN expression to activate AKT/GSK3β/β-catenin.** (A–D) AKT/GSK3β/β-catenin pathway protein level was tested. The levels of p-AKT Thr308, p-GSK3β Ser9, and β-catenin protein were notably declined after CORT treatment. They were significantly increased after miR-542-3p overexpression. After overexpression of PTEN or application of MK-2206, the levels of pathway proteins were declined. (E) The proliferation of HT-22 cells under various treatment conditions was tested by CCK-8. Compared with miR-542-3p overexpression alone, the cell viability was notably lower after the application of MK-2206. (F) LDH was used to detect the degree of damage in HT-22 cells under various treatment conditions. Compared with miR-542-3p overexpression alone, the degree of cell damage was markedly higher after the application of MK-2206. (G) The content of SOD was detected by ELISA. It was markedly diminished after the application of MK-2206. (H) The MDA content was detected by ELISA. The content of MDA was notably higher after MK-2206 was applied. (I and J) The level of ROS was tested by flow cytometry. ROS level was notably raised after the application of MK-2206. (K) IL-1β, TNF-α, and IL-6 levels were tested by ELISA. They were markedly expanded after the application of MK-2206 compared with miR-542-3p overexpression. (L and M) The apoptosis was tested by flow cytometry. After MK-2206 was applied, the apoptosis rate was obviously higher. *n* ═ 3, ^*^P < 0.05, **P < 0.01, ***P < 0.01. PTEN: Phosphatase and tensin homolog; GSK3β: Glycogen synthase kinase 3β; CCK-8: Cell counting kit-8; SOD: Superoxide dismutase; MDA: Malondialdehyde; ROS: Reactive oxygen species; IL: Interleukin; TNF-α: Tumor necrosis factor-α; ELISA: Enzyme-linked immunosorbent assay; NC: Negative control.

To investigate whether miR-542-3p affects nerve injury via the AKT/GSK3β/β-catenin pathway, we divided the experiment into four groups: the model group (mimics NC + vector), the miR-542-3p overexpression group (mimics + vector), the miR-542-3p overexpression + PTEN overexpression group (mimics + PTEN), and the miR-542-3p overexpression + AKT pathway inhibitor MK-2206 group (mimics + MK-2206). Following CORT treatment, the levels of p-AKT (Thr308), p-GSK3β (Ser9), and β-catenin proteins were significantly reduced (Figure 4A are 4B), suggesting that the AKT/GSK3β/β-catenin pathway plays a protective role in neuronal cell injury. However, miR-542-3p overexpression led to a significant increase in these pathway protein levels, indicating that miR-542-3p activates the AKT/GSK3β/β-catenin pathway. Conversely, PTEN overexpression or treatment with the AKT pathway inhibitor MK-2206 reduced the levels of these proteins (Figure 4C are 4D), demonstrating that both PTEN overexpression and AKT inhibition can counteract the pathway activation induced by miR-542-3p overexpression. In summary, miR-542-3p promotes AKT/GSK3β/β-catenin pathway activation by targeting and downregulating PTEN.

To verify the effect of the AKT/GSK3β/β-catenin pathway on neuronal cell injury, we found that MK-2206 significantly decreased cell viability (Figure 4E), increased LDH activity (Figure 4F), reduced SOD content (Figure 4G), elevated MDA levels (Figure 4H), and enhanced ROS intensity (Figure 4I and 4J). Additionally, MK-2206 significantly increased inflammatory factor levels (Figure 4K) and apoptosis rates (Figure 4L and 4M). These findings indicate that AKT pathway inhibition significantly counteracted the protective effects of miR-542-3p overexpression on neuronal cell injury. Furthermore, based on our experimental results related to the AKT/GSK3β/β-catenin pathway, we suggest that miR-542-3p may target and downregulate PTEN, thereby activating the AKT/GSK3β/β-catenin pathway and mitigating CORT-induced neuronal cell injury. This protective mechanism appears to be associated with the suppression of oxidative stress and inflammatory responses.

### miR-542-3p attenuates hippocampal neuronal damage in CORT-induced depressive mice

**Figure 5.**
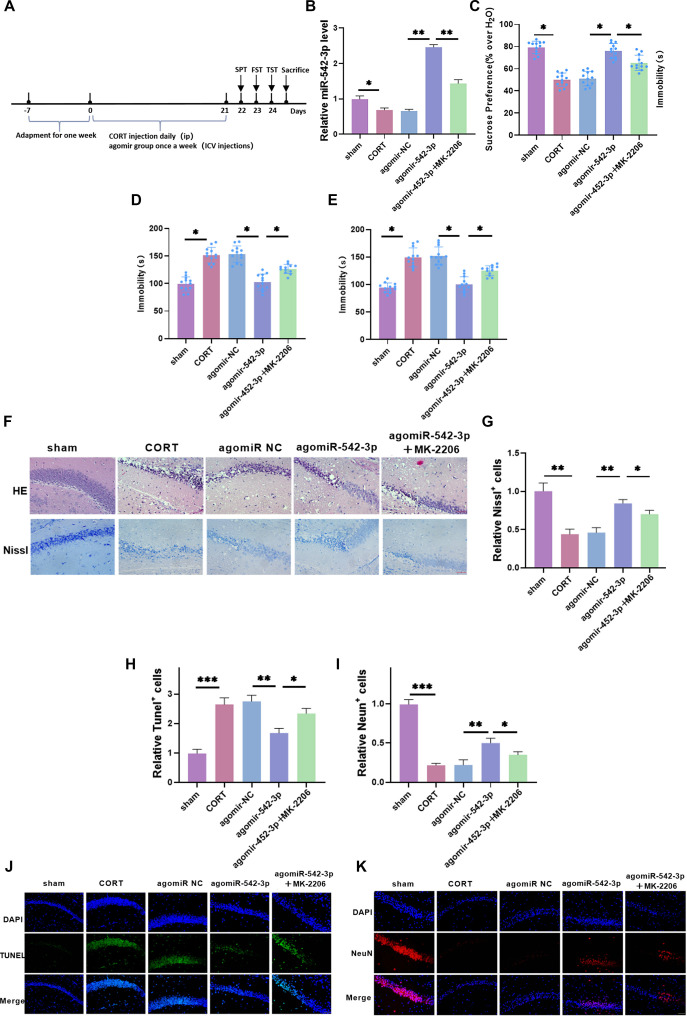
**miR-542-3p attenuates hippocampal neuronal damage in CORT-induced depressive mice.** (A) Experimental flow chart; (B) *miR-542-3p* level in the hippocampus of mice was tested by qRT-PCR. It was significantly raised after injection of agomiR-542-3p, indicating that it was effectively highly expressed *in vivo*; (C–E) The changes of depressive behavior in mice were evaluated by SPT, TST, and FST. The sucrose preference rate of mice was significantly decreased after CORT treatment, and the time of staying still in water and suspension was significantly raised. After overexpressed miR-542-3p, the depressive behavior was notably improved, but after application of MK-2206, the depressive behavior was significantly aggravated; (F and G) HE staining of hippocampal sections showed that after CORT treatment, nerve cells were swollen, irregular in shape, and a large amount of necrosis occurred. Neuronal necrosis declined after overexpressed miR-542-3p, but increased significantly after application of MK-2206. Nissl staining of hippocampal sections showed that Nissl body numbers declined significantly after CORT treatment, and increased significantly after overexpressed miR-542-3p, but decreased notably after application of MK-2206 (×40, 50 µm); (H–K) The apoptotic cells increased significantly, and NeuN positive cells decreased significantly after CORT treatment. After overexpressed miR-542-3p, apoptotic cells lessened significantly, and NeuN positive cells increased significantly, but the application of MK-2206 was reversed (×40, 50 µm). *n* ═ 5, *P* < 0.05, **P < 0.01, ***P < 0.01. CORT: Corticosterone; TST: Tail suspension test; HE: Hematoxylin-eosin; SPT: Sucrose preference test; FST: Forced swimming test; NC: Negative control.

Based on the *in vitro* function and mechanisms of miR-542-3p, we further investigated whether it could influence hippocampal neuronal damage in CORT-induced depressive mice. To test this, we administered intracerebroventricular injections of agomiR NC, agomiR-542-3p, and MK-2206, as illustrated in Figure 5A, followed by relevant assessments. After agomiR-542-3p injection, *miR-542-3p* levels significantly increased, while MK-2206 treatment led to a marked decrease (Figure 5B), confirming that *miR-542-3p* was effectively overexpressed *in vivo* and suitable for further experiments. Behavioral observations revealed that CORT treatment significantly reduced the mice’s sucrose preference rate and increased immobility time in both the forced swim and tail suspension tests (Figure 5C–5E), confirming the successful establishment of the depression model. Overexpression of miR-542-3p significantly alleviated depressive behaviors, whereas MK-2206 administration exacerbated them, indicating that miR-542-3p plays a role in improving depressive symptoms. Next, hippocampal neuronal damage was assessed using HE and Nissl staining. Following CORT treatment, nerve cells appeared swollen and irregular, with extensive necrosis and disrupted Nissl body morphology. Additionally, Nissl body count was significantly reduced (Figure 5F and 5G), neuronal apoptosis was elevated, and neuronal survival rate declined (Figure 5H–5K), indicating substantial neural tissue damage. However, miR-542-3p overexpression mitigated neuronal necrosis, restored Nissl body count, reduced apoptosis, and improved neuronal survival. In contrast, MK-2206 treatment exacerbated neural tissue damage. In conclusion, miR-542-3p alleviates hippocampal neuronal damage in CORT-induced depressive mice, suggesting its potential neuroprotective role.

### miR-542-3p attenuates hippocampal neuronal damage in CORT-induced depressive mice by regulating PTEN to activate AKT/GSK3β/β-catenin pathway

**Figure 6.**
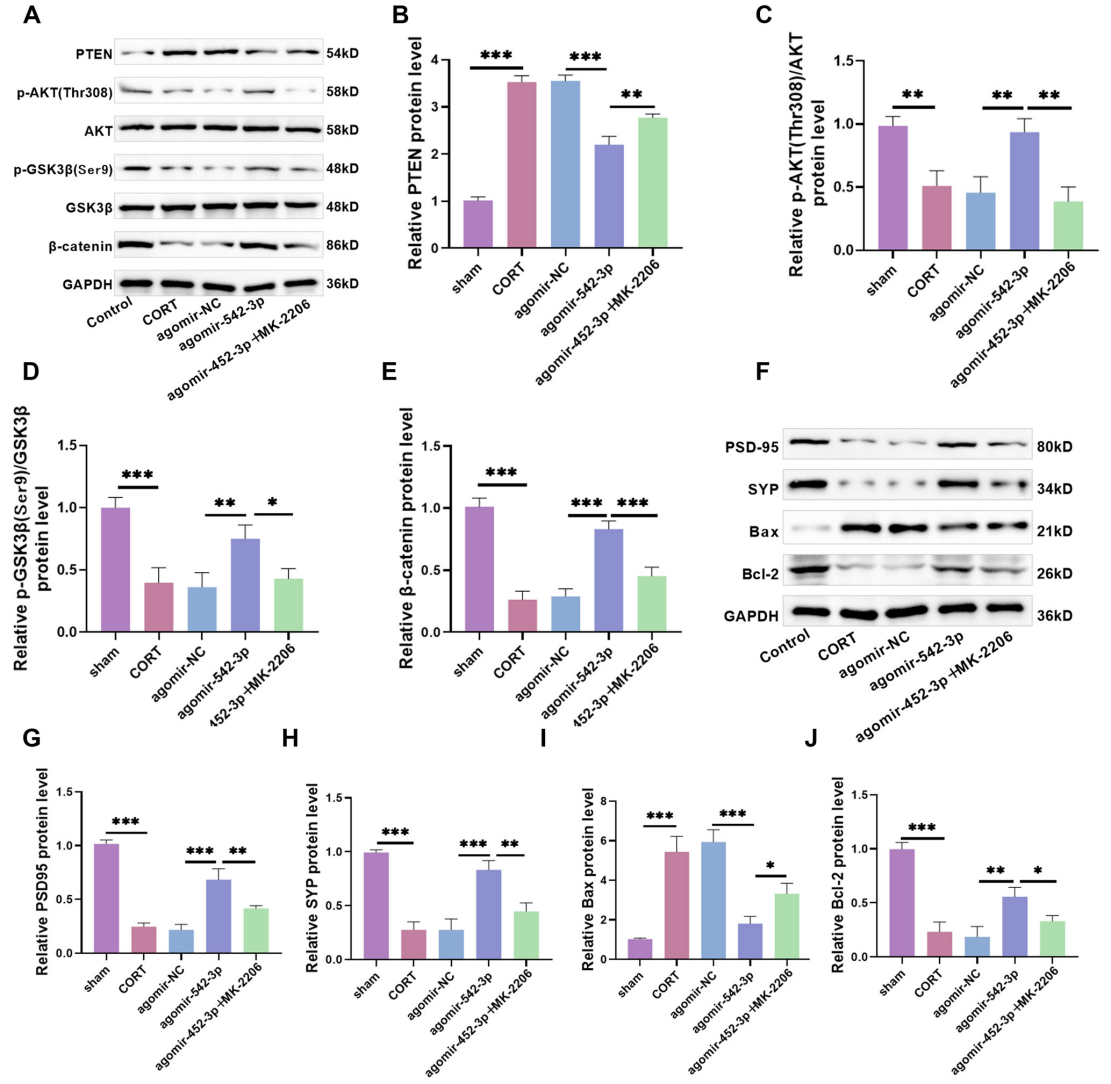
**miR-542-3p attenuates hippocampal neuronal damage in CORT-induced depressive mice by regulating PTEN to activate AKT/GSK3β/β-catenin pathway.** (A–E) The level of AKT/GSK3β/β-catenin pathway proteins were tested. It was found that the levels of p-AKT Thr308, p-GSK3β Ser9, and β-catenin protein were notably declined after CORT treatment. They was significantly increased after miR-542-3p overexpression. After the application of MK-2206, pathway protein levels were diminished; (F–J) The apoptosis and neuroplasticity-related protein levels were tested. Bax/Bcl-2 protein level enhanced significantly after CORT treatment, and the levels of SYP and PSD95 protein decreased significantly. After overexpressed miR-542-3p, Bax/Bcl-2 level was notably shrunk, and SYP and PSD95 protein levels were notably diminished, but the protein level was significantly reversed after application of MK-2206. *n* ═ 5, ^*^P < 0.05, **P < 0.01, ***P < 0.01. PTEN: Phosphatase and tensin homolog; Bcl-2: B-cell lymphoma-2; Bax: Bcl2-associated X protein; GSK3β: Glycogen synthase kinase 3β; CORT: Corticosterone; SYP: Synaptophysin; PSD95: Postsynaptic density 95; NC: Negative control.

Given that the AKT/GSK3β/β-catenin pathway can mitigate CORT-induced neuronal cell damage *in vitro*, we further investigated its effects *in vivo*. The trend in protein level changes was consistent with the *in vitro* results (Figure 6A–6E). Additionally, we examined apoptosis- and neuroplasticity-related proteins and found that Bax/Bcl-2 levels significantly increased following CORT treatment, while SYP and PSD95 levels significantly decreased. Overexpression of miR-542-3p led to a significant reduction in Bax/Bcl-2 levels and a significant increase in SYP and PSD95 levels. However, these effects were markedly reversed upon MK-2206 treatment (Figure 6F–6J), indicating that miR-542-3p mitigates CORT-induced neuronal damage by reducing apoptosis and promoting neural remodeling.

In summary, miR-542-3p downregulates PTEN, activating the AKT/GSK3β/β-catenin pathway to suppress apoptosis, enhance neural plasticity, and reduce CORT-induced neuronal damage.

## Discussion

Depression is a mental disorder characterized by emotional distress, reduced verbal expression, lack of pleasure, and low motivation. With the increasing pace of life and growing competitive pressures, the prevalence of depression has risen rapidly [26]. The occurrence of depression is linked to genetic, environmental, psychological, and social factors [27]. Its pathological mechanisms involve central nervous system inflammation, neuronal apoptosis, pyroptosis, and autophagy, along with atrophy and dysfunction in the cerebral cortex, white matter, amygdala, and hippocampus [28, 29]. However, many commonly used clinical drugs have significant adverse effects [3], highlighting the need for novel therapeutic options. Studies have shown that elevated levels of miR-542-3p can mitigate neuronal damage in epilepsy patients, reduce seizure frequency, and decrease neuronal apoptosis [8]. This suggests that miR-542-3p may also have potential in treating depression by protecting against neuronal damage. To investigate the underlying mechanisms, we used a CORT-induced neuronal injury model to examine the effects of miR-542-3p and its targets on neuronal injury after modeling.

In this study, CORT treatment significantly reduced cell viability while increasing cell damage and apoptosis. Overexpression or knockdown of miR-542-3p respectively increased or decreased cell viability and reduced or exacerbated cell injury and apoptosis. These findings suggest that miR-542-3p regulates the process of CORT-induced nerve injury, playing a protective role by enhancing cell viability and reducing apoptosis. Neuroinflammation and oxidative stress have been proposed as potential contributors to mental illness [30], as they can damage neurons. To investigate this, we analyzed oxidative stress and inflammatory markers. Following CORT treatment, SOD levels significantly decreased, while MDA, ROS, and inflammatory markers increased, accompanied by a marked rise in apoptosis. Overexpression or knockdown of miR-542-3p respectively restored or further reduced SOD levels, decreased or elevated MDA, ROS, and inflammatory markers, and reduced or exacerbated apoptosis. In summary, inflammatory responses and oxidative stress are involved in CORT-induced nerve injury, and miR-542-3p mitigates this damage by inhibiting oxidative stress and inflammation.

We used bioinformatics to analyze potential targets of *miR-542-3p* and identified *PTEN* and *APBB2*. Subsequent experiments showed that PTEN was the more effective target, so we focused on it for further study. Bioinformatics analysis revealed a binding site between miR-542-3p and PTEN, suggesting that miR-542-3p can directly target PTEN. PTEN has been shown to exacerbate sevoflurane-induced hippocampal cell apoptosis by inhibiting the MEK1/ERK pathway [13] and plays a crucial role in regulating neuronal regeneration. Its deletion or inhibition has been linked to enhanced axon growth in various neuronal populations [31]. However, whether miR-542-3p can mitigate nerve injury by targeting PTEN remains unclear. To investigate this, we overexpressed PTEN in the presence of miR-542-3p overexpression. This significantly reduced cell viability, increased injury and apoptosis, decreased SOD levels, and elevated MDA, ROS, and inflammatory factor levels. These findings suggest that PTEN overexpression exacerbates CORT-induced nerve injury and counteracts the protective effects of miR-542-3p overexpression. Combined with our bioinformatics results, this indicates that miR-542-3p may reduce nerve injury by targeting PTEN.

PTEN plays a crucial role in mediating oxidative stress, inflammation, autophagy, and neuroprotection by regulating AKT signaling activation. Silencing PTEN can stimulate AKT/mTOR signaling, reducing autophagy and oxidative stress levels, which in turn alleviates acute kidney injury in mice [32]. Additionally, PTEN inhibition promotes the proliferation of brain microvascular endothelial cells and enhances angiogenesis, exerting neuroprotective effects in focal cerebral ischemia in rats [33]. Beyond its role in autophagy and oxidative stress, PTEN is also a key inflammatory regulator. By modulating AKT signaling activity, it influences both inflammation and apoptosis. Inhibiting PTEN expression enhances AKT phosphorylation [34], while downregulating PTEN and activating AKT signaling reduce the production of inflammatory cytokines. This mechanism has been shown to protect mice from cerebral ischemia-reperfusion injury (CIRI) by mitigating inflammation and neuronal apoptosis [35]. AKT promotes GSK3β phosphorylation, thereby inhibiting GSK3β activity [36], stabilizing β-catenin, and facilitating its nuclear translocation to participate in nerve regeneration [37]. In this study, we examined the AKT/GSK3β/β-catenin pathway and found that CORT treatment significantly decreased the protein levels of p-AKT (Thr308), p-GSK3β (Ser9), and β-catenin. This suggests that activation of the AKT/GSK3β/β-catenin pathway may mitigate CORT-induced neuronal injury. Overexpression of miR-542-3p significantly increased pathway protein levels, indicating that miR-542-3p activates this pathway. However, when PTEN was co-overexpressed with miR-542-3p, pathway protein levels decreased, suggesting that PTEN overexpression weakens the activation induced by miR-542-3p. MK-2206, an AKT inhibitor, reduces AKT phosphorylation and its downstream target GSK3β while increasing β-catenin levels [38]. When MK-2206 was added in the context of miR-542-3p overexpression, protein levels declined. Furthermore, MK-2206 treatment significantly reduced cell viability, increased injury and apoptosis, lowered SOD levels, and markedly elevated MDA, ROS, and inflammatory factors. These findings further support that miR-542-3p targets PTEN to activate the AKT/GSK3β/β-catenin pathway, thereby reducing CORT-induced neuronal injury through the suppression of oxidative stress and inflammation. Moving forward, we plan to conduct additional experiments—such as β-catenin knockdown, overexpression, or localization studies—to further clarify the essential role of the AKT/GSK3β/β-catenin pathway in neuronal viability. Additionally, we will explore other known inhibitors and activators of this pathway to deepen our understanding of its neuroprotective mechanisms.

Finally, we verified the impact of miR-542-3p on depression *in vivo*. Depression was induced using CORT, and the model was evaluated through the SPT, TST, and FST. After modeling, the sucrose preference rate decreased, while immobility time in the TST and FST increased, indicating anhedonia and behavioral despair, confirming the successful establishment of the depression model.

Overexpression of miR-542-3p alleviated depressive behaviors by increasing sucrose preference, significantly reducing immobility time, enhancing self-help behavior, and decreasing hopelessness, suggesting its antidepressant effects. Previous studies have linked hippocampal neuronal damage to depression pathogenesis [39]. Thus, we examined hippocampal neurons and found that after CORT treatment, nerve cells appeared swollen and irregularly shaped, with extensive necrosis, a significant reduction in Nissl bodies, increased neuronal apoptosis, and a marked decline in survival rate—indicating severe neural damage. Overexpression of miR-542-3p notably alleviated these injuries, suggesting its neuroprotective role in CORT-induced depression. Furthermore, the AKT/GSK3β/β-catenin pathway protein expression was consistent with *in vitro* findings, reinforcing that miR-542-3p activates this pathway to mitigate neuronal damage. Since Bcl-2 inhibits apoptosis by protecting nerve cells, while Bax promotes apoptosis by binding to Bcl-2 and neutralizing its effects [40, 41], the Bax/Bcl-2 ratio serves as an indicator of apoptotic activity—the lower the ratio, the stronger the anti-apoptotic effect. Additionally, synaptic membrane proteins SYP and PSD95 influence synaptic neurotransmission and plasticity [42]. Following CORT induction, the Bax/Bcl-2 ratio increased, while SYP and PSD95 levels decreased, indicating weakened anti-apoptotic ability and impaired neural plasticity. Overexpression of miR-542-3p significantly reduced the Bax/Bcl-2 ratio and restored SYP and PSD95 levels. However, these effects were reversed upon MK-2206 treatment, confirming that miR-542-3p reduces CORT-induced neuronal damage by inhibiting apoptosis and promoting neural remodeling. In summary, miR-542-3p downregulates PTEN to activate the AKT/GSK3β/β-catenin pathway, thereby reducing apoptosis, repairing hippocampal neuronal damage, restoring neural structure and function, and alleviating CORT-induced depression in mice.

## Conclusion

This paper investigates the role of miR-542-3p in CORT-induced nerve injury. miR-542-3p modulates the AKT/GSK3β/β-catenin pathway by targeting PTEN expression, thereby reducing ROS and inflammatory factors while enhancing antioxidant enzyme activity. By inhibiting oxidative stress and the inflammatory response, miR-542-3p mitigates CORT-induced damage and apoptosis in HT-22 cells, highlighting its potential for neuronal protection. This study identifies miR-542-3p as a promising therapeutic target for nerve injury in depression, contributing to advancements in depression treatment. Previous studies have shown that miR-542-3p is significantly downregulated in the serum of patients with acute myocardial infarction and in epithelial ovarian cancer tissues. Additionally, it has been differentially expressed in various cancers, underscoring its potential clinical relevance. However, further research is needed to explore miR-542-3p levels and target factors in patients with nerve injury, as well as its predictive value for disease severity and prognosis.

## Data Availability

The data supporting the findings of this study can be obtained from the corresponding author, upon request.
